# A Case of Mohs Micrographic Surgery for Facial Porocarcinoma in an Adolescent and Literature Review

**DOI:** 10.1155/crom/9929153

**Published:** 2026-04-03

**Authors:** Wenwen Jing, Weiqi Lei, Zhicheng Gong, Pingxiu He, Yong Ai, Junwu Yang

**Affiliations:** ^1^ Department of Dermatologic Surgery, Dermatology Hospital of Jiangxi Province, Nanchang, Jiangxi, China; ^2^ Department of Dermatologic Surgery, Jiangxi Provincial Clinical Research Center For Skin Diseases, Nanchang, Jiangxi, China; ^3^ Department of Pathology, Candidate Branch of National Clinical Research Center for Skin Diseases, Nanchang, Jiangxi, China; ^4^ Department of Pathology, JXHC Key Laboratory of Skin Infection and Immunity, Nanchang, Jiangxi, China; ^5^ Department of Dermatologic Surgery, The Affiliated Dermatology Hospital of Nanchang University, Nanchang, Jiangxi, China

**Keywords:** adolescent, Mohs micrographic surgery, porocarcinoma

## Abstract

**Introduction:**

Eccrine porocarcinoma (EPC), the most common malignant tumor of the eccrine sweat glands, accounts for approximately 0.005%–0.01% of all skin malignancies. However, a standard treatment for EPC is currently lacking. Many previous studies demonstrated that it mainly occurs in the elderly and is extremely rare in adolescents. Here, we report a case of Mohs micrographic surgery for facial porocarcinoma in an adolescent patient.

**Case:**

A 19‐year‐old male adolescent was admitted to the hospital with a 6‐month history of a gradually enlarging itchy brown mass in the right nasolabial sulcus. After a pathological examination of the skin biopsy in the outpatient department, he was admitted to the hospital for Mohs surgery. Postoperatively, the wound defect was repaired using a local flap.

**Results:**

The patient recovered well after the surgery, and no recurrence was observed after 35 months of follow‐up.

**Conclusion:**

Our case demonstrated the effective early diagnosis and surgical intervention of EPC.

## 1. Introduction

Eccrine porocarcinoma (EPC), also known as malignant poroma of the eccrine sweat glands, is the most common malignant adnexal tumor. EPC originates from the intraepidermal portion of the excretory duct of the sweat glands and accounts for approximately 0.005%–0.01% of all skin malignancies [1]. Erythema and purple nodular lesions, the most common clinical manifestations, include purple or erythematous polypoid plaques that may be asymptomatic or associated with itching, ulceration, and spontaneous bleeding; these are less common and can persist for weeks to months [2]. According to recent studies, EPC primarily affects older adults with a mean age ranging from 63.6 to 65.6 years old [3] and is extremely rare in adolescents. Here, we report the case of a 19‐year‐old male adolescent patient and emphasize the typical characteristics and treatment approaches for this rare disease.

## 2. Case Presentation

A 19‐year‐old male student came to our hospital 6 months prior complaining of a brown mass on his right nasolabial fold that had been progressively enlarging and causing itching. The lesion was initially suspected as a suppurative granuloma. A pathological investigation was conducted in the outpatient department following a horizontal excision. The pathological examination revealed superficial scabbing and erosion, epidermal thickening and hyperplasia, a tumor in the dermis attached to the epidermis, clusters of basal‐like cells, disordered cellular architecture, cellular atypia, and prominent mitotic figures. No significant necrosis or palisades were observed, but a tumor originating from the epithelium was noted. Complete removal and submission for further histological and immunohistochemical analyses were recommended. The patient sought further surgical treatment and was admitted to our department on June 28, 2022.

Special examination revealed a 1‐cm–diameter brown mass in the right nasolabial fold. A small black crust was observed on the surface. Removal of the crust revealed mild erosion and ulceration with no obvious exudation. The boundary was clear and mobility acceptable, and no redness or swelling of the surrounding tissues was noted. No enlarged lymph nodes were observed in the head or neck.

Routine preoperative examinations including routine blood tests, C‐reactive protein level, liver and kidney function, blood glucose level, electrolyte levels, coagulation function, six male tumor markers, four infections, and routine electrocardiography were not significantly abnormal. A color Doppler ultrasound revealed two enlarged lymph nodes (one on either side of the neck). The lymph node on the right side (23.4 × 5.5 mm) was larger than that on the left side (19.5 × 5.5 mm). The lymph node hilum was absent, the medulla had mildly strong echogenicity, whereas the entire lymph node had low echogenicity.

On June 29, 2022, the patient underwent Mohs micrographic surgery. The intraoperative frozen section results indicated that no tumor tissue was present at the resection margin or base. The wound was then sutured directly. On July 4, 2022, a routine postoperative pathological examination showed tumor tissues at Margins 1 and 2 but no tumor tissues at Bases 1 and 2. Immunohistochemistry confirmed a diagnosis consistent with EPC, and no infiltration of lymph, blood vessels, and peripheral nerves was found. After an explanation was provided to the patient and their family, another Mohs surgery was performed on July 6, 2022. Perioperatively, a local extensive resection was performed. Intraoperative frozen sections indicated no tumor tissue at the resection margin, and the wound was repaired with a local skin flap. On July 8, 2022, a postoperative pathological examination indicated clean resection margins. The patient was discharged on July 10, 2022.

## 3. Pathological Manifestations

Hematoxylin and eosin staining (Figure 1) revealed hyperkeratosis, parakeratosis, superficial crusting, a roughly normal epidermis, a hyperplastic epithelial cell mass in the dermis, round or oval nuclei, eosinophilic or basophilic cytoplasm, atypia, duct‐like structures, and scattered inflammatory cell infiltration dominated by lymphocytes and eosinophils around the mass. Immunohistochemical staining revealed the following: cytokeratin (+), cytokeratin 7 focal (+), androgen receptor (−), epithelial membrane antigen (EMA) focal (+), Ber‐ep4 focal (+), and ki67 (+, 30%). Combined with the immunohistochemistry results, these findings were consistent with a diagnosis of EPC.

Figure 1HE showed hyperkeratosis, parakeratosis, superficial crusting, normal epidermis, hyperplastic epithelial cell mass in dermis, round or oval nucleus, eosinophilic or alkaline cytoplasm, atypia, visible duct‐like structure, and scattered lymphocytes, and eosinophils around the mass. Infiltration of inflammatory cells (a, HE 10×). Immunohistochemical staining showed CK7 positive (b, 10×).

**Figure:**
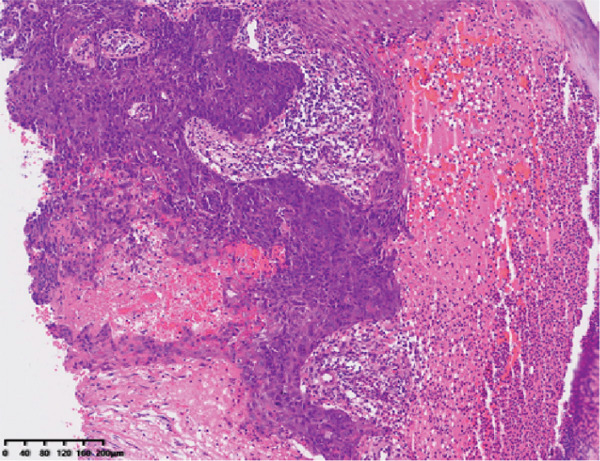
(a)

**Figure:**
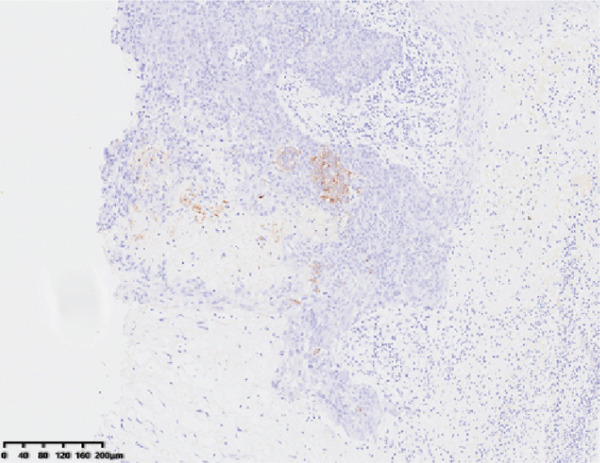
(b)

**Figure 2:**
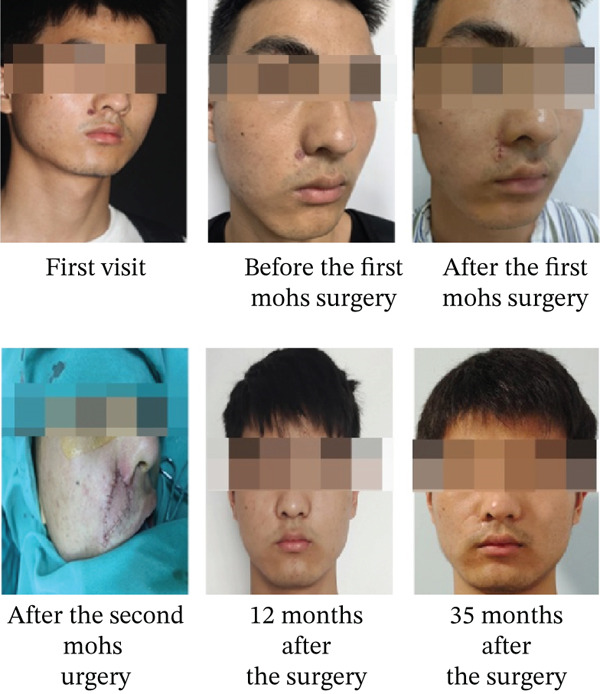
Preoperative and postoperative photos of the patient.

Postoperatively, the incision healed well, and no recurrence was observed after 35 months. The patient remains under follow‐up. Preoperative and postoperative clinical photographs of the patient are shown in Figure 2.

## 4. Discussion

This type of tumor was first described by Pinkus and Mehregan in 1963 [4]. Thereafter, in 1969, Mishima and Morioka, who coined the term EPC, were the first to describe the inner segment of the sweat duct of the apocrine sweat gland as the tumor′s origin [5]. The incidence of EPC is low, and it is more common in the elderly but rare in adolescents. The youngest patients reported to date included an 11‐year‐old by Le et al. [3], a 12‐year‐old by Urso et al. [6], and an 18‐year‐old by Liu et al. in whom no local recurrence but lymph node metastasis occurred during follow‐up [7]. Previously reported cases of adolescent and pediatric EPC are shown in Table 1. Our case report described EPC in a 19‐year‐old man in whom no local recurrence was noted after 35 months of follow‐up. The patient had enlarged cervical lymph nodes with regular lymph node morphology. The diagnosis was not confirmed histopathologically at the time of the visit, and the patient remains under follow‐up.

**Table 1:** Previously reported cases of EPC in adolescents.

Patient age (y)	Patient sex	Tumor location	Treatment	Follow‐up details
18	Male	Nasal dorsum [7]	Wide excision	Metastasis to the lymph nodes in the neck was noted. Radiotherapy combined with neck lymph node dissection was performed. Local recurrence occurred in the surgical area of the neck. Immunotherapy was subsequently administered.
11	Male	External ear [8]	Wide excision of the external ear canal, radical mastoidectomy, and skin graft reconstruction	—
8	Female	Left calf [9]	Wide excision	Ten months later, the patient remains well without recurrence or metastasis.
12	Female	Vulva [6]	Wide excision	The patient remains well without recurrence or metastasis.

However, the pathogenesis of EPC remains unclear. Previous studies implicated the tumor suppressor gene TP53 in its pathogenesis [10]. Moreover, other noteworthy mutant genes in EPC include *NCOR1*, *CDKN2A*, *GSK3B*, *EGFR*, and *PIK3CA* [11]. Recent studies demonstrated that EPC is primarily driven by somatic mutations affecting various pathways. Fusion gene mutations are the primary carcinogenic drivers, followed by somatic mutations that promote tumor progression. Fusion genes frequently involve members of the *PAK* gene family [12].

The clinical manifestations of EPC are diverse and the differential diagnosis extensive; therefore, a histopathological analysis is important for its diagnosis. Various histological patterns (e.g., squamous cells, clear cells, spindle cell differentiation, and melanocyte colonization) are observed in EPC tumor cells. Although EPC cells lack specific immune characteristics, immunohistochemical staining may help distinguish them from other tumors. The most common immunohistochemical markers were EMA, carcinoembryonic antigen, S‐100, CD117, and cytokeratin 19 [13]. Previous studies showed that EMA is designed to identify ductal structures. Riera‐Leal et al. and Perm et al. stated in their analysis of EPC that the diagnosis had relatively high sensitivity. The report of Shiohara et al. indicated positive EMA staining in all 11 EPC samples. However, Beer reported a 96% EMA positivity rate in skin squamous cell carcinoma specimens. Therefore, although the use of EMA to identify ductal structures can support the diagnosis of EPC, this detection also highlights the sweat ducts of squamous cell carcinoma, suggesting that EMA immunostaining cannot clearly diagnose EPC [14].

A standard treatment for EPC is currently lacking. Many previous studies demonstrated that Mohs surgery effectively treats skin adnexal cancer, including EPC [3]. Recent literature identified Mohs surgery and complete marginal assessments as the preferred treatment [1]. Previous studies demonstrated that two stages of Mohs surgery are required in most EPC cases to reach tumor clearance; some researchers used modified Mohs surgery and slow Mohs to treat EPC and confirmed a lack of recurrence during an average follow‐up of 20 months [15–17]. In our case, after the first Mohs surgery, the patient′s intraoperative frozen‐to‐normal pathological results suggested that the tumor tissue was visible at the resection margin; thus, a second Mohs surgery was performed to achieve tumor clearance. Therefore, based on the literature and our experience, Mohs surgery may be a treatment option for EPC. For cases involving special parts (e.g., the eyelids, nose, and nasolabial sulcus), slow Mohs surgery can be considered. The resected tissue is subjected to comprehensive margin assessments after paraffin embedding to ensure complete removal of the tumor tissue. Normal tissue should be preserved whenever possible to reduce the treatment effects on an individual′s appearance and function. However, the surgical time for paraffin embedding is long, which delays incision closure. Large‐scale controlled trials are needed to explore the best surgical method for treating EPC.

Studies have shown that approximately 20% of EPC patients will experience local recurrence, whereas another 20% will have metastasis. The regional lymph nodes were the most common sites of metastasis. Distant metastasis sites include the lungs, liver, and brain. The mortality rate of patients with lymph node metastasis is 67%, and most patients with distant metastasis have a poor prognosis [18]. In metastatic eccrine porocarcinoma (mEPC), a variety of chemotherapeutic drugs and small molecule targeted drugs have been used, but none have demonstrated long‐term benefits for mEPC. Therefore, there is an urgent need to develop novel systemic treatment strategies [19–21]. In our case, prior to treatment, we discovered that the cervical lymph nodes were enlarged, and an ultrasound examination showed suspicious features requiring fine‐ or hollow‐needle aspiration to determine the presence of lymph node metastasis. For various reasons, he did not undergo further examinations at that time; during the subsequent follow‐up process, no face‐to‐face consultation was conducted, meaning that the lymph nodes were not closely observed.

Sentinel lymph node status is a key indicator of malignant tumor staging. Positive sentinel lymph node status indicated that the tumor had already caused regional lymph node metastasis; thus, the risk classification was upgraded from early (Stages I–II) to late (Stage III), suggesting a poor prognosis. Our patient has been followed‐up for 20 months without local recurrence; however, his lymph node condition still requires close monitoring. In the future, we will closely monitor the lymph node condition, perform further examinations, and provide corresponding treatment if positive lymph nodes are found.

## 5. Study Limitations

The limitations of our study are that we neither clarified whether lymph node metastasis was present at the time of treatment nor regularly reviewed the lymph nodes during follow‐up. Moreover, our case represents a single instance and the study lacked a pathological examination of the lymph nodes. Therefore, the general applicability of our findings to treatment recommendations is limited.

## 6. Conclusion

EPC is a clinically challenging tumor, and our case demonstrates the efficacy of early diagnosis and surgical intervention. We hope to improve skin surgeons′ understanding of this rare tumor through case reports and studies to improve its diagnosis and treatment. Ultimately, such efforts will help us further explore and clarify the mechanism of EPC to enable the identification of new therapeutic targets and improvement of patient prognosis and quality of life.

## Author Contributions

Junwu Yang: concept and planning of the work described. Yong Ai approved the final submitted version of the manuscript. Wenwen Jing: acquisition, analysis and interpretation of the data, and drafting revision of the manuscript. Pingxiu He provided the data. Weiqi Lei and Zhicheng Gong: critical revision of the manuscript.

## Funding

No funding was received for this manuscript.

## Disclosure

All authors reviewed the manuscript. All authors have seen and approved the final version for publication.

## Ethics Statement

This study was approved by the appropriate ethics review board of our hospital and was in accordance with the Helsinki Declaration. The patient in this study has given written informed consent for publication for all clinical images and health information.

## Conflicts of Interest

The authors declare no conflicts of interest.

## Data Availability

Data sharing is not applicable to this article as no datasets were generated or analyzed during the current study.
